# Delivery of bFGF for Tissue Engineering by Tethering to the ECM

**DOI:** 10.1155/2015/208089

**Published:** 2015-10-11

**Authors:** Chawapun Suttinont, Yasumasa Mashimo, Masayasu Mie, Eiry Kobatake

**Affiliations:** Department of Environmental Chemistry and Engineering, Interdisciplinary Graduate School of Science and Engineering, Tokyo Institute of Technology, 4259 Nagatsuta, Midori-ku, Yokohama 226-8502, Japan

## Abstract

Delivery of growth factors to target cells is an important subject in tissue engineering. Towards that end, we have developed a growth factor-tethered extracellular matrix (ECM). Here, basic fibroblast growth factor (bFGF) was tethered to extracellular matrix noncovalently. The designed ECM was comprised of 12 repeats of the APGVGV peptide motif derived from elastin as a stable structural unit and included the well-known cell adhesive RGD peptide as an active functional unit. To bind bFGF to the ECM, an acidic amino acid-rich sequence was introduced at the C-terminus of the ECM protein. It consisted of 5 repeats of 4 aspartic acids and a serine, DDDDS. bFGF has a highly basic amino acid domain. Therefore, bFGF was tethered to the ECM protein by electrostatic interaction. Cells cultured on bFGF-tethered ECM were well attached to the ECM and induced proliferation without addition of soluble bFGF.

## 1. Introduction

Growth factors are important for regulating a variety of cellular processes, and they are indispensable for tissue engineering. To increase the local concentration of growth factors, several techniques for delivering growth factors have been investigated [1]. In many cases, growth factors are embedded in hydrogels for delivery. In addition, immobilization of growth factors to extracellular matrices (ECMs) has been developed for delivering growth factors to cells.

A strategy to immobilize growth factors to ECMs has advantages compared to the addition of soluble growth factors. When soluble growth factors are added to cells, it is difficult to control their local concentration due to diffusion, cell uptake, and degradation [2]. To overcome such problems, the strategy of tethering growth factors to artificial ECMs was developed. In those studies, growth factors have been immobilized to ECMs chemically or genetically [3–5]. In our previous study, growth factors were noncovalently immobilized on genetically engineered ECMs [6, 7]. For growth factor immobilization, helical peptides forming coiled-coil helical interactions were fused to growth factors and ECMs, respectively. Using this technique, we have developed a method to coimmobilize three different types of growth factors, basic fibroblast growth factor (bFGF), epidermal growth factor (EGF), and single-chain vascular endothelial growth factor (scVEGF_121_), onto an ECM protein in order to promote angiogenesis [7].

Basic fibroblast growth factor (bFGF) is a commonly used growth factor for tissue engineering because of its wide variety of functions. For example, it is a stimulator of proliferation, differentiation, and migration of multiple cell types [8]. It has a highly basic amino acid domain allowing it to directly interact electrostatically with the acidic region of another protein [9]. Therefore, we focused on the property of the basic region of bFGF for tethering to the designed ECMs.

**Figure 1:**
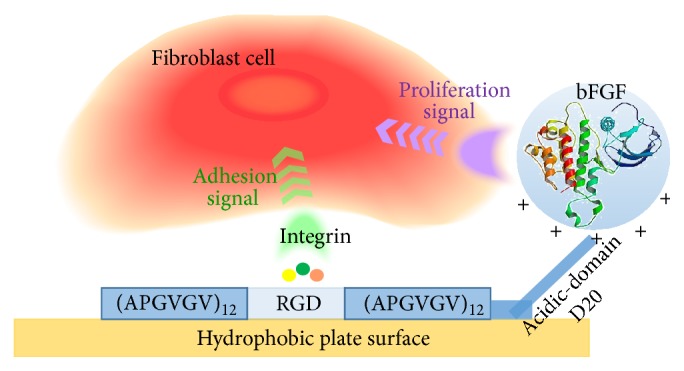
Schematic drawing of bFGF-tethered designed ECM through electrostatic interaction.

In this study, bFGF-tethered ECM was developed for the purpose of delivering growth factors to cells. For tethering bFGF, a polyaspartic acid domain (D20) was introduced to our designed artificial ECM, ERE, which consists of 12 repeats of the Ala-Pro-Gly-Val-Gly-Val (APGVGV) motif derived from elastin as a stable structural unit. The repeated APGVGV sequence is highly hydrophobic, allowing ERE to adsorb well onto the hydrophobic surface of the dish. It also included the well-known cell adhesive RGD sequence as an active functional unit [5]. Fusion proteins encoding D20 were shown to form a complex with a cationic polymer, polyethylenimine, by electrostatic interaction because the aspartic acid-rich domain is negatively charged under physiological conditions [10, 11]. Therefore, it was expected that bFGF would be tethered to D20 fused to ERE (ERE-D20) by electrostatic interaction between the basic rich domain of bFGF and D20 (Figure 1). Here, bFGF tethering to ERE-D20 and the cell adhesion activity of ERE-D20 were evaluated. Finally, cells were cultured on bFGF-tethered ERE-D20 and we examined the induction of cell proliferation activity.

## 2. Materials and Methods

### 2.1. Plasmid Construction

The plasmid pET-ERE constructed in our laboratory was digested with* Nco* I and* Bgl* II to obtain the ERE gene fragment [5]. The plasmid pET-His-C2D20 encoded 5 repeats of 4 aspartic acids and a serine, DDDDS (constructed as previously described) [11]. The plasmid was digested with* Nco* I and* Bgl* II followed by insertion of the ERE gene fragment. The resulting plasmid for expression of ERE-D20 protein in* E. coli* was named pET-His-ERE-(D4S)_5_.

### 2.2. Protein Expression and Purification

The constructed plasmid, pET-His-ERE-(D4S)_5_, was transfected into* E. coli* BL21(DE3) competent cells by heat shock. Transformed* E. coli* cells were cultured in Luria-Bertani (LB) medium with ampicillin at 37°C. Protein expression was induced by addition of 1 mM isopropyl *β*-D-1-thiogalactopyranoside (IPTG). Cells were cultured overnight at 30°C and harvested by centrifugation and resuspended in BugBuster Reagent with Benzonase Nuclease (Sigma-Aldrich). After 30 min rotation at 4°C, the sample was centrifuged. The supernatant was applied to HIS-Select Nickel Affinity Gel (Sigma-Aldrich) followed by incubation at 4°C for 1 h. After washing with phosphate buffer (50 mM sodium phosphate, 300 mM NaCl, pH 8.0), proteins were eluted by phosphate buffer with 100 mM imidazole. The resultant protein solution was dialyzed using Slide-A-Lyzer Dialysis Cassettes (Pierce) against phosphate-buffered saline (PBS). The purified protein was analyzed by 12% sodium dodecyl sulfate polyacrylamide gel electrophoresis (SDS-PAGE) and the protein concentration was measured with a BCA assay kit (Pierce).

### 2.3. Cell Culture

The murine fibroblast cell line C3H10T1/2, obtained from the Riken Cell Bank, was grown in Dulbecco's modified Eagle's medium (DMEM) with 10% fetal bovine serum (FBS) and antibiotics (100 U/mL penicillin, 100 *μ*g/mL streptomycin).

### 2.4. Adsorption of ERE-D20 on the Hydrophobic Surface of Plates

Solutions of ERE-D20 and ERE were added to 96-well suspension culture plates (Sumilon, MS-8096R) in varied concentrations and incubated for 2 h at 37°C with shaking. Plates were washed with PBS-T (PBS including 0.05% Tween 20) followed by blocking with Blocking One (Nacalai Tesque, Inc.) overnight at 4°C. After washing with PBS-T, anti-poly-histidine antibody (Sigma-Aldrich) was added to the plate and incubated for 1 h at 37°C followed by washing with PBS-T again. Then, anti-mouse IgG peroxidase conjugate (Sigma-Aldrich) was added and incubated for 1 h at 37°C. After washing with PBS-T, TMB peroxidase substrate (KPL, Inc.) was added to the plate. Finally, 1 M HCl was added to stop the reaction and the absorbance at 450 nm was measured by a microplate reader.

### 2.5. Cell Adhesion Activity of ERE-D20

Cell adhesion assays were performed in a 24-well suspension culture plate (Iwaki). Purified ERE-D20 and ERE proteins (1000 nM) were added to culture plates and incubated for 2 h at 37°C. Then, the plate was washed with PBS three times. C3H10T1/2 cells suspended in FibroLife Serum-Free Medium (Lifeline Cell Technology) were added to the wells (8000 cells/well) and cultured for 6 h. Attached cells were evaluated with a Cell Counting Kit-8 (Dojindo).

### 2.6. Tethering of bFGF to ERE-D20 through Electrostatic Interaction

The wells of a 96-well suspension culture plate (Sumilon, MS-8096R) were coated with ERE and ERE-D20 (100 or 1000 nM), respectively. After incubation for 2 h at 37°C with shaking, wells were washed with PBS-T followed by blocking with Blocking One overnight at 4°C. After washing with PBS-T, various concentrations of bFGF were added and plates were incubated for 1 h at 37°C. After washing with PBS-T, a solution of 1/1000 diluted rabbit anti-bFGF antibody (Sigma-Aldrich) was added and incubated for 1 h at 37°C. After washing with PBS-T, anti-rabbit IgG-HRP (Jackson ImmunoResearch Inc.) was added and reacted for 1 h at 37°C. After washing with PBS-T, TMB peroxidase substrate was added to the plate. Finally, 1 M HCl was added to stop the reaction and the absorbance at 450 nm was measured by a microplate reader.

### 2.7. Cell Proliferation on bFGF-Tethered ERE-D20

The wells of a 24-well suspension culture plate (Iwaki) were coated with ERE-D20 (1000 nM) and incubated for 2 h at 37°C. After washing with PBS three times, bFGF (100 nM) was added to the wells and the plate was incubated for 2 h at 37°C. After washing with PBS, C3H10T1/2 cells suspended in FibroLife Serum-Free Medium without recombinant bFGF were added to wells (8000 cells/well) and cultured. During culture, media were changed every 2 days. The number of cells on days 1 and 5 was evaluated using a Cell Counting Kit-8.

### 2.8. Statistical Analysis

Values are given as mean value ± standard deviation. Statistical analysis was performed by independent two-sample* t*-test with equal valiances. Values of *P* < 0.05 were considered to be statistically significant.

## 3. Results and Discussion

### 3.1. Design and Expression of ERE-D20 Protein

**Figure 2:**
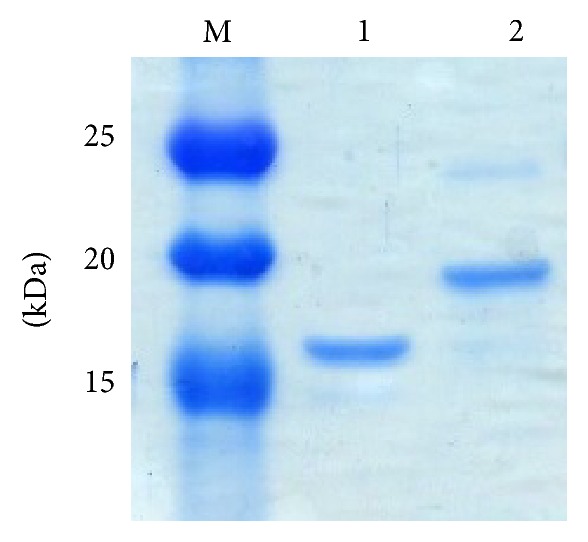
SDS-PAGE analysis of purified ERE and ERE-D20 proteins. Lane M, SDS Broad Range Marker; lane 1, ERE protein; and lane 2, ERE-D20 protein.

The designed extracellular matrix, ERE, was genetically fused with 5 repeats of 4 aspartic acids and serine for tethering bFGF via electrostatic interaction (ERE-D20). To express ERE and ERE-D20 proteins,* E. coli* BL21(DE3) was used as the host strain. Expressed proteins were purified by nickel affinity gel using His-tag. The molecular mass of ERE and ERE-D20 was 15.3 kDa and 18.3 kDa, respectively. The size and purity of samples were confirmed by SDS-PAGE (Figure 2). The band of ERE in SDS-PAGE sometimes appeared larger than the expected molecular size from its amino acid sequence. This behavior has been previously reported for ELP-fused proteins due to the physicochemical characteristics of ELPs [12]. However, the band of ERE-D20 appeared at nearly the expected molecular size. It is suggested that the physicochemical characteristics of the ELPs were cancelled by the negatively charged D20 domain.

### 3.2. Cell Adhesion Activity of ERE-D20

**Figure 3:**
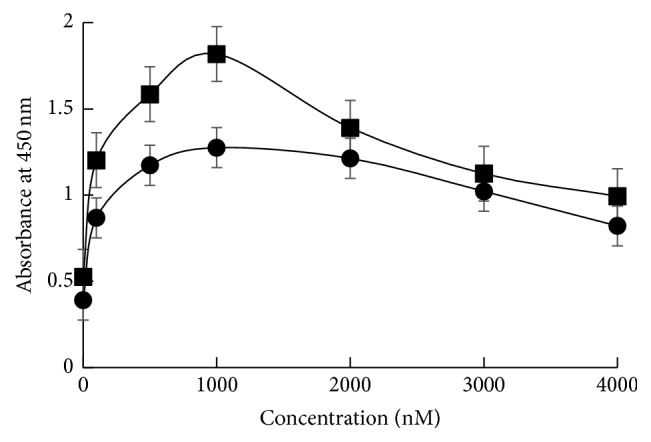
Adsorption of ERE-D20 on the hydrophobic surface of plates. Various concentrations of ERE protein (square) and ERE-D20 (circle) were coated on the surface of suspension culture plates, respectively. Error bars show the standard deviations of three independent measurements.

First, adsorption of ERE-D20 onto the hydrophobic surface of the dish was evaluated. After addition of proteins to 96-well suspension culture plates, adsorbed proteins were detected with an anti-poly-histidine antibody. As shown in Figure 3, adsorption of ERE-D20 was slightly lower than that of ERE. It would be caused by the hydrophilicity of D20 domain. However, it was shown that ERE-D20 maintained the ability to adsorb onto the hydrophobic surface of the dish, even after fusion with D20.

**Figure 4:**
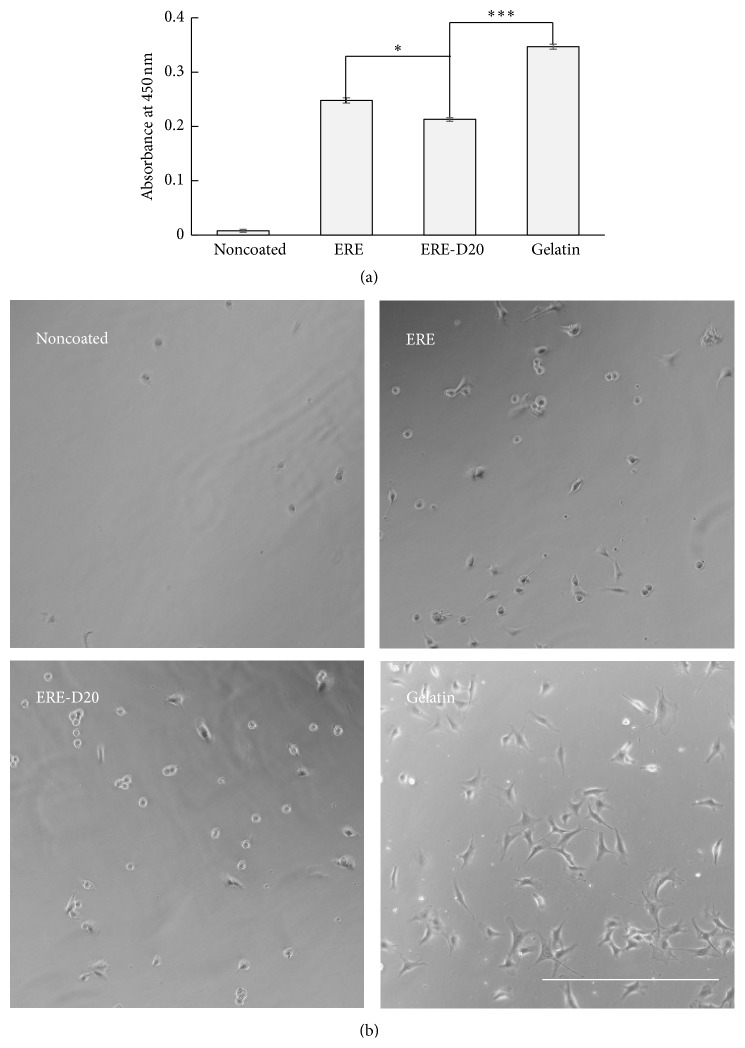
Cell adhesive activity of ERE-D20. (a) Evaluation of cell numbers after incubation for 6 h. Error bars show the standard deviations of three independent measurements. Statistically significant differences are indicated for *P* < 0.05 (*∗*) and *P* < 0.005 (*∗∗∗*). (b) Cells were cultured on noncoated plate, plate coated with ERE, ERE-D20, and gelatin. Scale bar = 500 *μ*m.

The ERE protein has a cell adhesion peptide, RGD, and it has shown cell adhesion activity. However, ERE-D20 has a negatively charged domain. Generally, cell surfaces are charged negatively. Therefore, the effects of D20 on cell adhesion activity were evaluated. Cells were seeded onto wells coated with ERE or ERE-D20 protein and incubated for 6 h. As shown in Figure 4, cells seeded into the noncoated wells did not attach. In contrast, cells seeded into the well coated with ERE-D20 were attached on the surface; the number was less than cells in the wells coated with gelatin. However, the number of cells in the well coated with ERE-D20 was almost the same as that with ERE. These results proved that ERE-D20 maintained cell adhesion activity, even after fusion with D20.

### 3.3. Tethering of bFGF to ERE-D20 through Electrostatic Interaction

**Figure 5:**
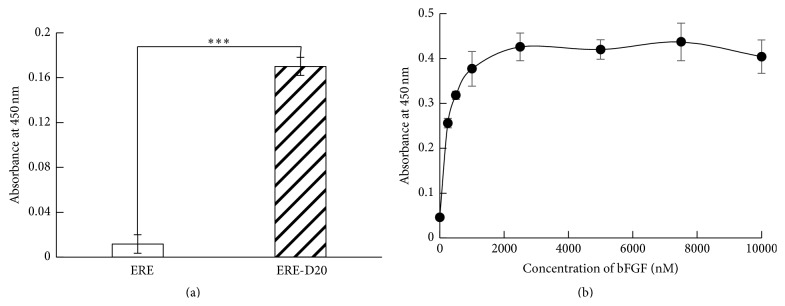
Evaluation of bFGF tethered to ERE-D20. (a) Specific binding between basic domain of bFGF and negatively charged D20 of ERE-D20 (100 nM). (b) Concentration dependency of bFGF tethered to ERE-D20 (1000 nM). Error bars show the standard deviations of three independent measurements. Statistically significant difference is indicated for *P* < 0.005 (*∗∗∗*).

The interaction between ERE-D20 and bFGF was determined by ELISA using anti-bFGF antibody. Recombinant bFGF was added to wells of 96-well suspension culture plates coated with 100 nM ERE or ERE-D20. The tethered bFGF was detected using anti-bFGF antibody. As shown in Figure 5(a), bFGF did not bind to the wells coated with ERE without D20. On the other hand, the wells coated with ERE-D20 showed a strong signal. Then, we evaluated the binding of varied bFGF concentrations on 1000 nM ERE-D20 (Figure 5(b)). The tethered bFGF increased in a concentration-dependent manner and saturated at 2500 nM. Previously, Suzuki et al. reported that fusion protein encoding D20 was shown to form complex with polyethylenimine by electrostatic interaction [10]. The calculated isoelectric points of ERE-D20 and bFGF are approximately 3.9 and 9.6, respectively. Therefore, ERE-D20 should be able to tether bFGF through electrostatic interaction under physiological condition. These results proved that bFGF was tethered to ERE-D20 through electrostatic interaction between the basic domain of bFGF and D20 of ERE-D20.

### 3.4. Cell Proliferation on bFGF-Tethered ERE-D20

**Figure 6:**
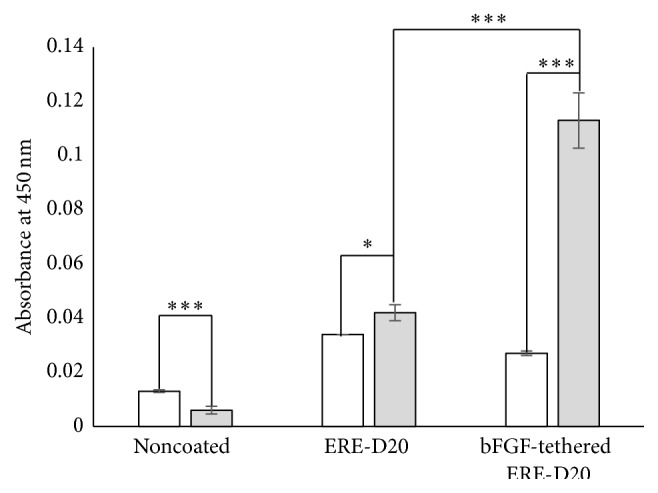
Induction of cell proliferation activities by culture on bFGF-tethered ERE-D20. Cells were cultured on ERE-D20 with or without tethered bFGF for 1 day (white bars) and 5 days (gray bars). Error bars show the standard deviations of three independent measurements. Statistically significant differences are indicated for *P* < 0.05 (*∗*) and *P* < 0.005 (*∗∗∗*).

Proliferation of cells cultured on bFGF-tethered ERE-D20 was evaluated. Cells were seeded into the wells of 24-well suspension culture plates coated with ERE-D20 alone and/or ERE-D20 tethered to bFGF. As shown in Figure 6, cells seeded onto the wells coated with ERE-D20 without tethered bFGF attached on the well surface, but cell growth was scarcely observed after 5 days of culture. On the other hand, when cells were seeded into wells coated with ERE-D20 tethered to bFGF, we observed significant proliferation at day 5. To confirm the effects of tethered bFGF, activation of MEK/ERK and JNK pathway should be evaluated with or without inhibitors for FGFR1. These experiments are under evaluation in our laboratory.

In our previous studies, growth factors were noncovalently immobilized on designed ECMs via helical peptides forming coiled-coil helical interaction [6, 7]. In that technique, growth factors are required to fuse helical peptide. It may have a risk to reduce activities of growth factors, while the present technique using electrostatic interaction does not need modification of growth factors. In principle, any negatively charged growth factors under physiological condition can be tethered to ERE-D20 without loss of activities. However, it would be also disadvantage of this method, since negatively charged proteins existing in serum would have effects on binding and release of growth factors. Now we are starting experiments to clarify these effects.

## 4. Conclusion

In this study, we showed that bFGF could be tethered to our designed extracellular matrix (ERE-D20) via electrostatic interaction between the basic domain of bFGF and the acidic domain of ERE-D20. Cells cultured on bFGF-tethered ERE-D20 were well attached on ERE-D20 and underwent proliferation without addition of soluble bFGF. From these data, bFGF tethering to an ECM via electrostatic interaction could be applied for delivery of growth factors in tissue engineering.
